# Temporal transcriptomic profiling identifies core regulators in cytokine-induced gut barrier disruption in differentiated Caco-2 cells

**DOI:** 10.1007/s00011-026-02288-5

**Published:** 2026-06-08

**Authors:** Hyo Shin Yoon, Shining Ma, Matt Kanke, Wan-Jung Wu, Uyen Hoang, Jessica A. Tan, Alex Wilks, Priyanka Patel, Sundeep Chandra, Xin Luo, Daniel Lu, Scott Martin, Mark Wilson, Menno Van Lookeren Campagne, Chi-Ming Li, Cheng-Yuan Kao

**Affiliations:** 1 Inflammation Therapeutic Area, Amgen Research, South San Francisco, CA 94080 USA; 2 Discovery Sciences, Advanced Technologies, & AI, Amgen Research, South San Francisco, CA 94080 USA; 3 Translational Safety & Risk Sciences, Amgen Research, South San Francisco, CA 94080 USA; 4 Amgen R&D Postdoctoral Fellows Program, South San Francisco, CA 94080 USA

**Keywords:** Intestinal epithelial barrier, Time series transcriptome, Rho GTPase, Tight junction, *HNF4A*, *SLC26A3*, Inflammatory bowel disease

## Abstract

**Objective:**

The intestinal epithelial barrier maintains gut homeostasis, and its disruption contributes to inflammatory diseases such as Inflammatory Bowel Diseases (IBD). Although barrier restoration is a promising therapeutic strategy, most current approaches target immune responses rather than epithelial function. Since barrier injury and repair progress through discrete phases, this study aimed to define time-resolved epithelial transcriptional programs and identify candidate regulatory nodes for barrier-focused therapeutic intervention.

**Methods:**

Differentiated Caco-2 epithelial monolayers were used to model cytokine-induced barrier disruption and profiled by time-resolved bulk RNA-seq. Transcriptional dynamics and candidate regulators were identified using pairwise differential expression, likelihood-ratio testing, pathway enrichment, and upstream-regulator inference. Candidate nodes were tested by pharmacologic perturbation, and disease relevance was assessed by qPCR and histology in human IBD colon tissues.

**Results:**

Time-resolved profiling revealed dynamic epithelial programs involving the Rho GTPase cycle, sirtuin signaling, non-canonical WNT signaling, and junction/EMT-associated pathways. Upstream-regulator inference highlighted JAK2, TNF, gasdermin D, and β-estradiol, and pharmacologic perturbation of these nodes mitigated cytokine-induced barrier loss. HNF4A emerged as a gut epithelial transcriptional hub regulating targets including SATB2 and SLC26A3. Notably, SLC26A3 was markedly reduced in IBD colon tissues.

**Conclusions:**

This study provides a time-resolved epithelial framework and highlights therapeutic opportunities for barrier-focused interventions.

**Supplementary Information:**

The online version contains supplementary material available at 10.1007/s00011-026-02288-5.

## Introduction

The gastrointestinal epithelial barrier is central to mucosal and systematic homeostasis, enabling selective nutrient uptake while restricting passage of luminal antigens, pathogens, and other potentially harmful macromolecules [1]. Disruption of intestinal barrier contributes to a wide spectrum of conditions including inflammatory diseases, infection, drug-induced injury, ischemia, and post-surgical complications [2].

Inflammatory bowel diseases (IBD), including ulcerative colitis (UC) and Crohn’s disease (CD), are chronic, relapsing-remitting disorders characterized by dysregulated immune responses and compromised intestinal barrier integrity [3,4]. Current therapeutic strategies predominantly focus on immunosuppression, targeting cytokine signaling pathways and leukocyte recruitment [5,6]. While agents such as Upadacitinib, a Janus kinase (JAK) inhibitor, have demonstrated clinical efficacy in UC with a reported 48% risk reduction in relapse [7], pharmacologic interventions aimed at restoring intestinal epithelial barrier function remain absent. Notably, increased intestinal permeability has been observed in a subset of healthy first-degree relatives of patients with CD, suggesting it may serve as a preclinical marker and risk factor for disease onset [8,9]. In patients with CD, elevated intestinal permeability has also been associated with risk of relapse [10]. However, the underlying mechanisms driving these alterations in barrier function are still not fully understood.

Epithelial barrier integrity is primarily governed by dynamic tight junction (TJ) complexes localized at the apical region of polarized epithelial cells. These structures are composed of key transmembrane proteins such as claudins, occludin, and cytoplasmic scaffolding proteins like zonula occludens-1 (ZO-1). Although originally viewed as passive barriers, tight junctions are now recognized as dynamic signaling hubs [11]. For example, Claudin-2, a pore-forming TJ protein, increases paracellular permeability to cations and is upregulated in inflamed tissues under the influence of proinflammatory cytokines such as IL-13 and TNF-α, contributing to diarrhea and barrier disruption in IBD [12]. Conversely, occludin overexpression enhances epithelial barrier function in vitro, representing a potential protective response [13]. Interestingly, casein kinase 2 (CK2), which phosphorylates occludin is reported to weaken its interaction with ZO-1 and enhance claudin-2 expression [14]. The inhibition of CK2 has been shown to restore barrier function and attenuate colitis in T cell-mediated murine models [14]. ZO-1, a multifunctional peripheral membrane protein, anchors claudins and occludin to the cytoskeleton and interacts with numerous other proteins, including ZO-2/3, cingulin, JAM-A, and F-actin [15] and deletion of intestinal epithelial ZO-1 increases macromolecular permeability and disrupts apical membrane structure, both in vitro and in vivo [16].

Another key regulator of perijunctional dynamics is myosin light chain kinase (MLCK), which increases permeability via phosphorylation of myosin light chain (MLC), leading to contraction of the perijunctional actomyosin ring (PAMR) and tension on TJ proteins [17,18]. Inhibition of MLCK by novel small molecules, such as divertin, has been shown to preserve barrier integrity by preventing TNF-induced MLC phosphorylation and occludin endocytosis, effectively reducing diarrheal diseases [19]. Moreover, inhibition of MLCK interacting protein, tacrolimus-binding protein (FKBP8) reported to prevent MLCK1 recruitment to intracellular junctions and downstream events [20].

In parallel, the hypoxia-inducible factor (HIF) pathway also plays a critical role in barrier regulation. Under normoxic conditions, prolyl hydroxylase domain proteins (PHD1 and PHD2) promote HIF1α degradation. Stabilization of HIF1α via PHD inhibition enhances expression of barrier-protective genes involved in mucus production, TJ formation, and wound healing [21]. In preclinical models, oral administration of PHD-specific inhibitors restored barrier function and ameliorated colitis by stabilizing HIF1α [22], identifying the HIF pathway as a potential barrier target for therapeutic intervention.

Rho GTPases have emerged as key regulators of intestinal epithelial homeostasis through their control of tight junction assembly and actin cytoskeleton dynamic [23]. These processes are essential for maintaining epithelial barrier integrity and regulating paracellular permeability and lack of RhoA in IECs in mice increases intestinal permeability and inflammation [24,25]. Disruption of Rho GTPase signaling has been shown to impair junctional organization, resulting in increased intestinal permeability and facilitating the translocation of luminal antigens and microbes, thereby promoting mucosal inflammation [23,24].

Beyond their role in epithelial barrier function, Rho GTPases are critical modulators of immune cell behavior. They regulate fundamental processes such as leukocyte migration, phagocytosis, and lymphocyte activation, thereby shaping both innate and adaptive immune responses [26]. Importantly, genetic studies have identified multiple IBD susceptibility loci that intersect with Rho GTPase regulatory pathways, further underscoring their relevance in disease pathogenesis [23].

To date, differentiated Caco-2 human colon adenocarcinoma cell line culture remains a widely adopted, reductionist system for dissecting epithelial-intrinsic control of permeability despite their cancer-derived origin and lack of three-dimensional physiological context [27]. Polarized Caco-2 monolayers cultured on transwells and differentiated into enterocyte-like monolayers, expressing tight junctions, brush border, ionic transporter, digestive enzymes and various receptors (e.g., vitamin B12, vitamin D3, epidermal growth factor receptor (EGFR)) as well as sugar transporters (GLUT1-5, SGLT1) and a range of immune related proteins (IL-6, IL-8, TNF-α, TGF-β, etc.) resembling the physiological properties of the human intestinal epithelium enable standardized, scalable investigation of barrier regulation and compound testing [28].

Although Caco-2 barrier models are widely used, genome-scale analyses under cytokine challenge have largely been limited to single-timepoint readouts or microarray co-cultures [29], leaving a gap in epithelium-only, time-course RNA-seq data. We address this gap by profiling differentiated Caco-2 monolayers across a cytokine-induced barrier-disruption series, resolving dynamic epithelial programs and actionable control points.

In this study, we modeled gut epithelial barrier failure by challenging differentiated Caco-2 monolayers with cytokines and profiling the response over time by bulk RNA-seq. A joint analysis that integrates pairwise differential expression with a likelihood-ratio test across the series resolves transient and nonlinear programs, centering the Rho GTPase cycle as the nexus of cytoskeletal and tight-junction remodeling. Network inference points to tractable upstream regulators such as *JAK2*, gasdermin D, *TNF*, and β-estradiol and to HNF4α as an epithelial transcriptional hub with downstream genes such as *SATB2* and *SLC26A3*. Perturbing these upstream regulators attenuates barrier loss, echoing the activity of approved *JAK* and *TNF* inhibitors and underscoring the value of a reductionist, epithelial-first platform for mapping mechanisms and prioritizing barrier-directed interventions.

## Methods

### Cell culture

The human colorectal adenocarcinoma cell line Caco-2 expressing Cas9 was purchased from UBIGENE Biosciences Co., Ltd (Guangzhou, China). Cells were maintained in 77% minimum essential media (Gibco) supplemented with 20% heat inactivated fetal bovine serum (Sigma Aldrich), 1% Glutamax, 1% Sodium pyruvate, 1% non-essential amino acids (Gibco) and 100 µg/ml hygromycin (Gibco) in a humidified atmosphere (37 °C and 5% CO_2_). For differentiation, cells were seeded at 8 × 10^4^ cells/200µl on 0.4 μm pores polyester HTS transwell membrane (Corning-Costar, Acton, MA) and grown as monolayers. After 12 days, recombinant human TNF-α and human IFN-γ were treated to both apical and basal chamber for 0 h (*N* = 4 per group), 6 h (*N* = 7 ~ 8 per group), 9 h (*N* = 7 per group), 18 h (*N* = 4 per group), 30 h (*N* = 8 per group) and 54 h (*N* = 8 per group). Cytokines were added once at the start of treatment, and the medium was not replenished during the 54-hour exposure. The cells were cultured and used for experiments within fewer than 12 passages to ensure consistent differentiation status.

For 3D microfluidic model system, OrganoReady Colon Caco-2 (Mimetas BV, The Netherlands) comprising 64 ready-to-use Caco-2 seeded against collagen I was used.

### Recombinant cytokines, reagents and cell viability

Recombinant human TNF-α (HZ-1014; Proteintech) and recombinant human IFN-γ (285-IF/CF; Bio-techne) were purchased. Cell viability was determined using CellTiter-Glo (G7573, Promega, Leiden, the Netherlands) according to the manufacturer’s protocol. This kit detects viable cells by quantifying ATP which indicates the presence of metabolically active cells. The assay was performed in a 96-well plate format and luminescence was quantified using Tecan microplate reader.

### Barrier integrity assay

Trans-Epithelial Electrical resistance (TEER) was measured and calculated using an epithelial volt-Ohm meter (EVOM2; World Precision Instruments, Sarasota, FL). The TEER of empty transwell was subtracted. TEER measurements for Organoplates were performed using an automated multichannel impedance spectrometer (OrganoTEER, Mimetas BV).

### FITC-dextran intestinal permeability assay

Cells were seeded onto Transwell inserts (0.4-µm pore size) and maintained under standard culture conditions until a confluent, polarized monolayer was established. Prior to the assay, monolayers were washed with pre-warmed Hank’s balanced salt solution (HBSS). FITC-dextran diluted in HBSS was added to the apical compartment (250 µl; final concentration 1 mg/ml), and 800 µl fresh HBSS was added to the basolateral compartment. The cells were incubated at 37 °C for 20 min. Basolateral medium was collected for analysis. Fluorescence intensity was measured using a Tecan microplate reader (excitation 485 nm, emission 528 nm).

### RNA isolation and gene expression analysis

Total RNA was extracted using the RNeasy Mini Kit (Qiagen) according to the manufacturer’s instructions and 800ng of RNA was reverse transcribed with cDNA synthesis master mix (Legene Biosciences, CA, USA). TaqMan assay probes are purchased from ThermoFisher, and the assay number of each probe was listed in the Table S1. RT-PCR was performed using QuantStudio™ 7 Flex (Applied Biosystems). Fold change in expression was calculated using 2-ΔΔCT method using GAPDH as house-keeping gene and normalized with untreated control.

### Bulk RNA sequencing

**Fig. 1:**
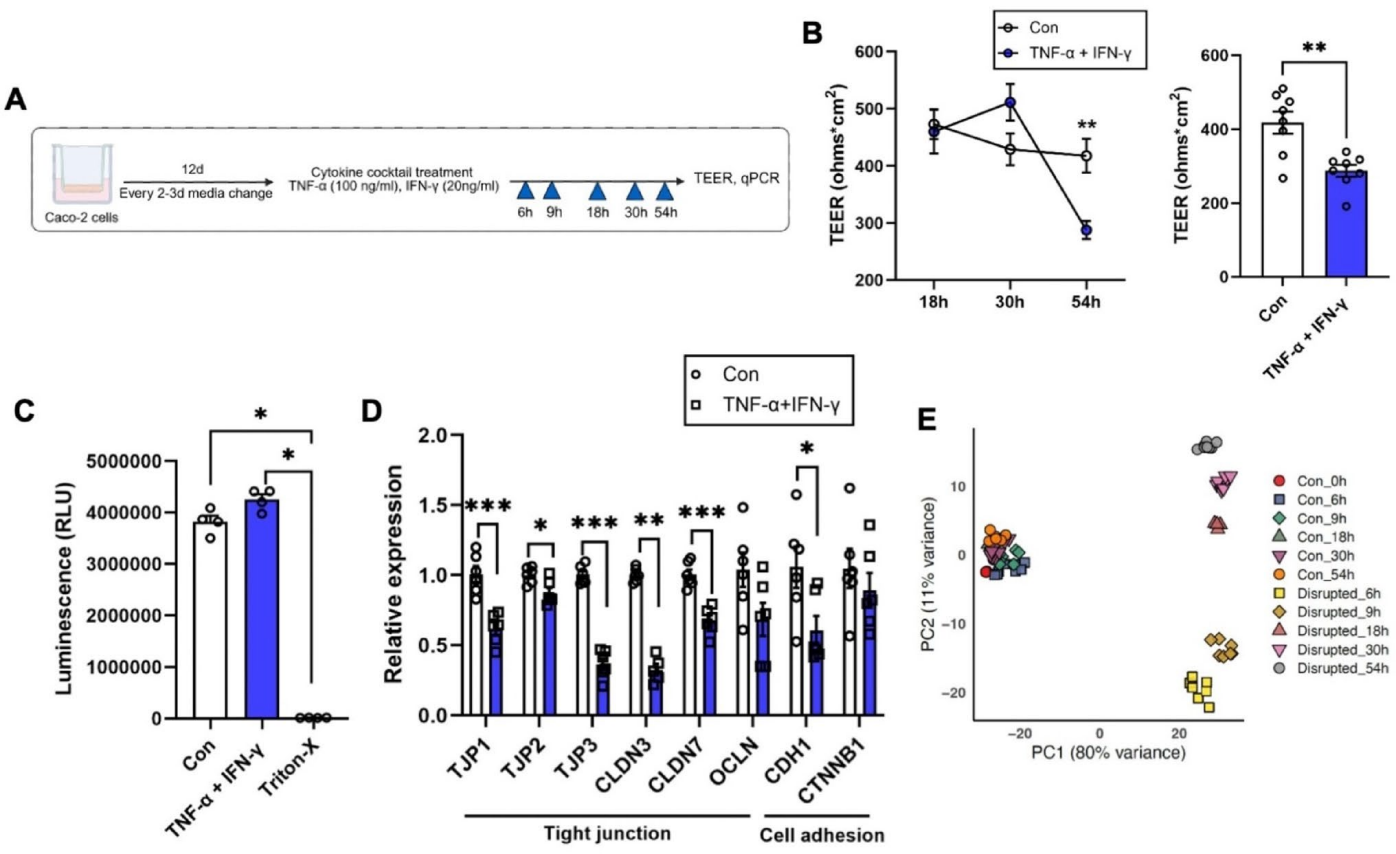
Cytokine induced intestinal barrier disruption in Caco-2 cells and transcriptome analysis. **A** Overview of experimental design using cytokine mediated intestinal barrier disruption model. **B** Gut barrier integrity using TEER measurement at 18 h, 30 h and 54 h and endpoint at 54 h. *N* = 8 /group. **C** Cell viability test at 54 h. *N* = 4/group. **D** Relative expression of tight junction and cell adhesion related genes by qPCR *N* = 6 /group. **E** PCA plot of differentially expressed genes across each time point: 0 h (*N* = 4 per group), 6 h (*N* = 7 ~ 8 per group), 9 h (*N* = 7 per group), 18 h (*N* = 4 per group), 30 h (*N* = 8 per group) and 54 h (*N* = 8 per group). Bulk RNA-seq and qPCR data were derived from two independent experiments. Other assays were performed in a single experiment with technical independent wells per condition. The number of replicates is indicated for each experiment. Data are presented as means ± SEM. *p-*values were determined by an ANOVA test with Sidak’s multiple comparison test (B, left), Mann-Whitney U test was used for B (right) and C. Unpaired t-test was used for D. *p < 0.05, **p < 0.01, ***p < 0.001, and ****p < 0.0001

The concentration and quality of the isolated RNA samples were assessed and determined on Agilent 4200 TapeStation system. Quality of RNA was checked using the Bioanalyzer. RNA extracted from cells with a RIN (RNA integrity number) score of 7 was considered for further analysis. Sequencing libraries were constructed using the TruSeq Stranded mRNA Library Preparation Kit (Illumina) and IDT for Illumina-TruSeq RNA UD Indexes. Libraries were sequenced on a NovaSeqX to a minimum depth of 35 million reads. Raw sequencing reads were aligned to the human genome reference (Human.B38) using the OSA aligner (Qiagen Omicsoft). Aligned reads were collapsed into gene-level counts using Omicsoft’s RSEM implementation and the genomic locations in the GENCODE v33 gene model. Data analysis was performed using the Ingenuity Pathway Analysis (IPA) package (Qiagen) (Fig. 1).

### Pairwise DE analysis

Pairwise differential expression analyses were performed using DESeq2, applying significance thresholds of false discovery rate (FDR) < 0.05 and |fold change| > 1.5 to identify differentially expressed genes (DEGs) between control (Con) and disrupted (Disrupted) conditions at each time point. Prior to analysis, genes with fragments per kilobase of transcript per million mapped reads (FPKM) values less than 1 were excluded to remove lowly expressed transcripts, and only protein-coding genes were retained for downstream analyses.

Volcano plots were generated in R (tidyverse, ggplot2, ggrepel) to visualize the differential expression profiles of protein-coding genes between cytokine treated and undisrupted Caco-2 cells at five time points (6, 9, 18, 30, and 54 h), as shown in Supplementary Fig. 1A.

**Fig. 2:**
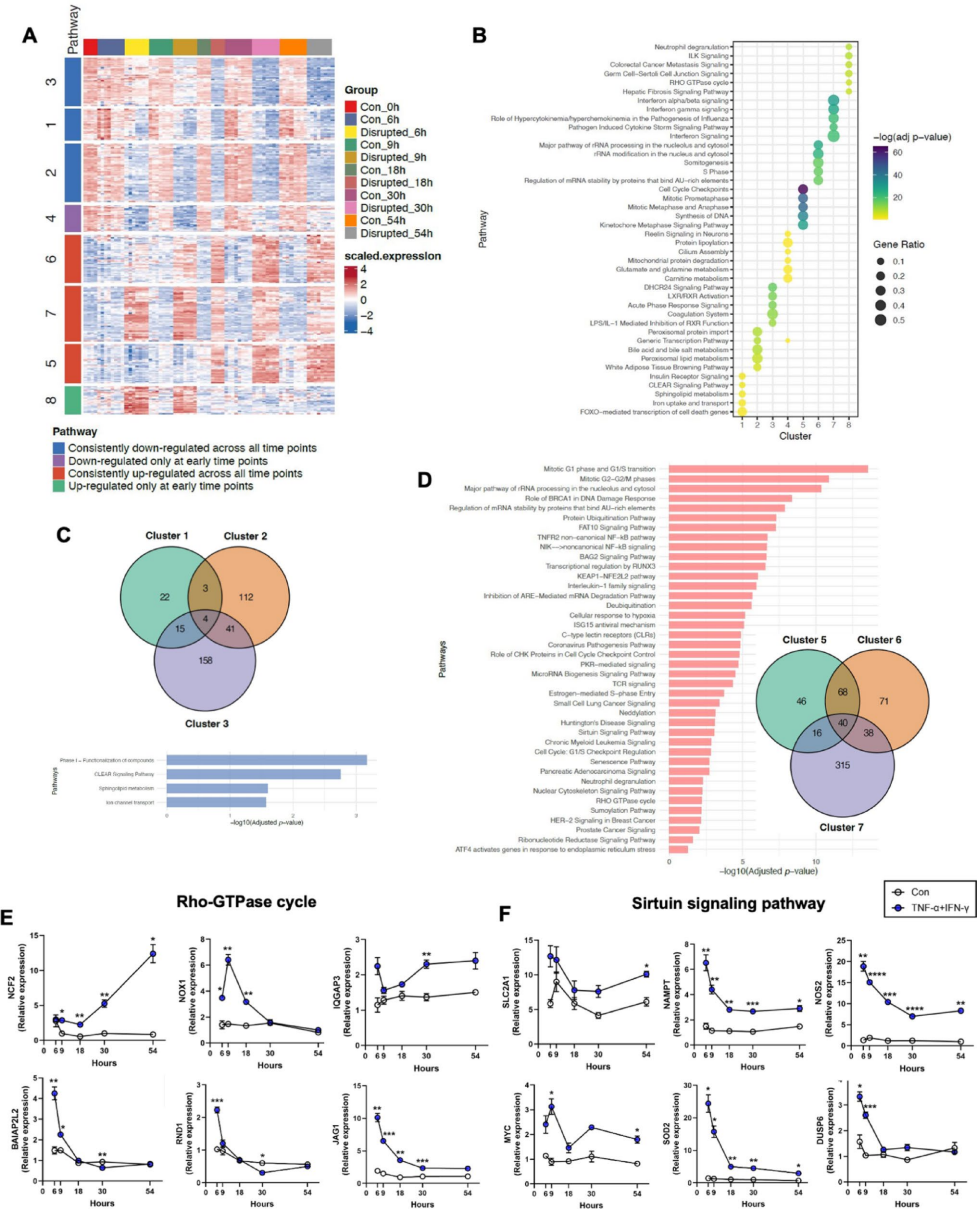
Pairwise differential gene expression analysis and enriched pathway analysis in differentiated Caco-2 cells following cytokine exposure at each time point. RHO GTPase cycle and Sirtuin Signaling pathway are selected from overlapping pathways induced by cytokine exposure and DEGs are validated. **A** Heatmap with hierarchical clustering of differentially expressed genes at different time points (0 h, 6 h, 9 h, 18 h, 30 h, 54 h) after the cytokine treatment in Caco-2 cells. **B** Top 5 enriched IPA pathways in pairwise DEG clusters in each cluster and the significance is represented as -log 10 (adjusted *p*-value) Some of the pathways share the same significances and captured more than 5. **C** Venn diagram showing significantly overlapping enriched pathways among cluster 1–3, with core overlapping pathways. **D** Venn diagram of overlapping enriched pathways among clusters 5–7, with the core overlapping pathways. **E** and **F** Validation of the DEGs levels by qPCR in selected overlapping pathways in Caco-2 cells (*N* = 4 /group). qPCR data were derived from two independent experiments. Data are presented as means ± SEM. *p-*values were compared to control at each time point and determined by an ANOVA test with Sidak’s multiple comparison test (D and E). *p < 0.05, **p < 0.01, ***p < 0.001, and ****p < 0.0001

The union of DEGs identified across all pairwise comparisons throughout the time course was subjected to hierarchical clustering to group genes exhibiting similar temporal expression dynamics into eight distinct clusters. These clusters were visualized in a clustered heatmap to illustrate dynamic gene expression patterns (Fig. 2A). To elucidate the biological significance of each cluster, Ingenuity Pathway Analysis (IPA) was performed to identify enriched canonical pathways and biological processes associated with the transcriptional programs of Caco-2 cells.

### Temporal clustering (LRT analysis)

To examine and model dynamic gene expression changes over time, a likelihood ratio test (LRT) was performed using the DESeq2 package. The full model included both Time and Treatment factors along with their interaction (design = ~ Time + Treatment + Treatment: Time), capturing the combined effects of treatment conditions and temporal progression. To specifically assess the contribution of time-dependent variation beyond treatment effects, a reduced model (reduced = ~ Treatment + Time) was employed, effectively removing the interaction term (Treatment: Time) from the design. This comparison between the full and reduced models allowed for identification of genes whose expression profiles were significantly influenced by the interaction between treatment and time, highlighting temporal regulatory effects driven by experimental conditions.

**Fig. 3:**
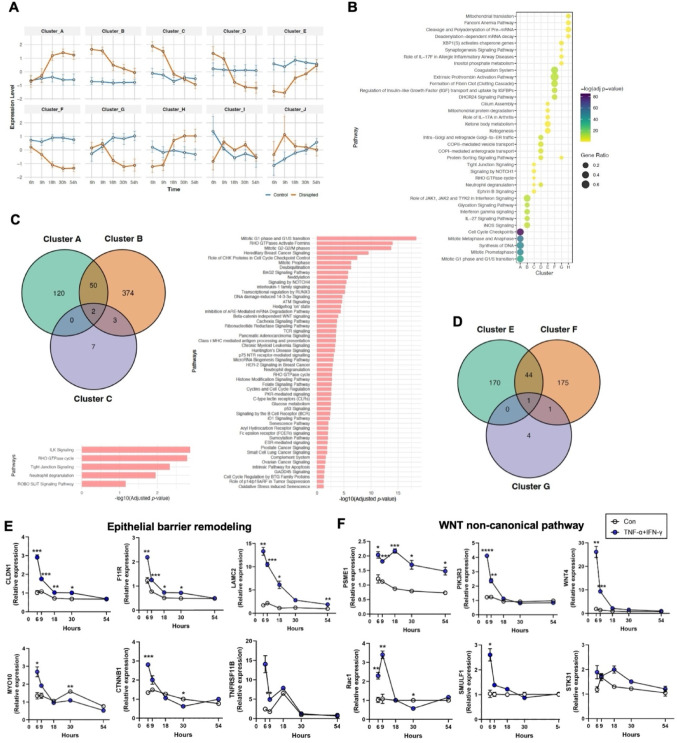
LRT analysis and enriched pathway analysis in differentiated Caco-2 cells with cytokine exposure at each time point. Tight junction signaling network and WNT non-canonical pathway are selected among the overlapped pathways induced by cytokine exposure and DEGs are validated. **A** LRT analysis of the cytokine induced barrier disrupted Caco-2 cells, identifying 10 distinct temporal clusters. **B** Top 5 enriched pathway analysis of each cluster based on biological processes with pathway significance represented as -log 10 (adjusted P-value). **C** Venn diagram showing significantly overlapping enriched pathways among cluster 1–3 and the core overlapping pathways are shown between cluster 1 and 2 and between cluster 2 and 3. **D** Venn diagram of significantly overlapping enriched pathways among clusters 5–7 and the core overlapping cluster between cluster 5 and 7. (E and F) Validation of the DEGs levels by qPCR in selected overlapping pathways in Caco-2 cells. *N* = 4 /group. qPCR data were derived from two independent experiments. Data are presented as means ± SEM. *p*-values were determined by an ANOVA test with Sidak’s multiple comparison test for E and F. *p < 0.05, **p < 0.01, ***p < 0.001, and ****p < 0.0001

Genes showing statistically significant differences (adjusted *p* < 1e − 10) from the LRT were retained as time-responsive candidates. The expression counts of these genes were then variance-stabilized using regularized log transformation (rlog) for downstream visualization. Subsequently, temporal expression patterns were clustered using the degPatterns function from the DEGreport package, grouping genes with similar time-dependent trajectories. These clusters were visualized as smoothed line plots (Fig. 3A) showing median expression and interquartile ranges across time points for each treatment. This approach enabled the delineation of distinct temporal expression profiles, revealing coordinated transcriptional responses and dynamic regulatory programs across treatment conditions.

### Enrichment pathway analysis of DEGs

Pathway enrichment analysis was performed using Ingenuity Pathway Analysis (IPA, QIAGEN, Redwood City, www.qiagen.com/ingenuity) to identify biological pathways and molecular functions significantly associated with genes derived from both pairwise differential expression (DE) and temporal LRT (Likelihood Ratio Test) analyses. For the pairwise DE analysis, genes showing significant expression differences between treatment conditions at each time point were identified using DESeq2 and subsequently analyzed in IPA to determine condition-specific pathway enrichment. In parallel, the temporal LRT analysis-based on the model ~ Time + Treatment + Treatment: Time with the reduced model ~ Treatment + Time-was applied to capture genes with significant time-dependent expression changes or treatment-time interactions.

The significant genes from each analysis were uploaded into IPA, and canonical pathway enrichment was assessed using adjusted p-values to determine significance. The top enriched pathways for each gene cluster or comparison were visualized using bubble plots, where the − log₁₀(adjusted p-value) represented pathway significance and the gene ratio indicated the proportion of differentially expressed genes contributing to each pathway. This integrative approach enabled comparison of condition-specific and temporally regulated biological responses, providing a comprehensive view of the molecular mechanisms underlying dynamic gene expression changes across time and treatment conditions. All the enriched pathways in each cluster analyzed by both methods are shown in **supplementary data 1 and 2.**

### Upstream regulators analysis

To identify molecules upstream molecules (i.e. transcription factors, drugs, compounds, cytokines, miRNAs) in the literature-complied QIAGEN Knowledge Base whose activity could potentially explain the observed gene expression changes, we used IPA analysis. The P-value (determined with Fisher’s exact test) reflects the significance of the overlap between the regulated sets within the dataset. A Z-score could not be analyzed due to the cluster-based analysis. For validation test, the list of the chemicals/drugs were used and shown in **Table ****S2**.

**Fig. 4:**
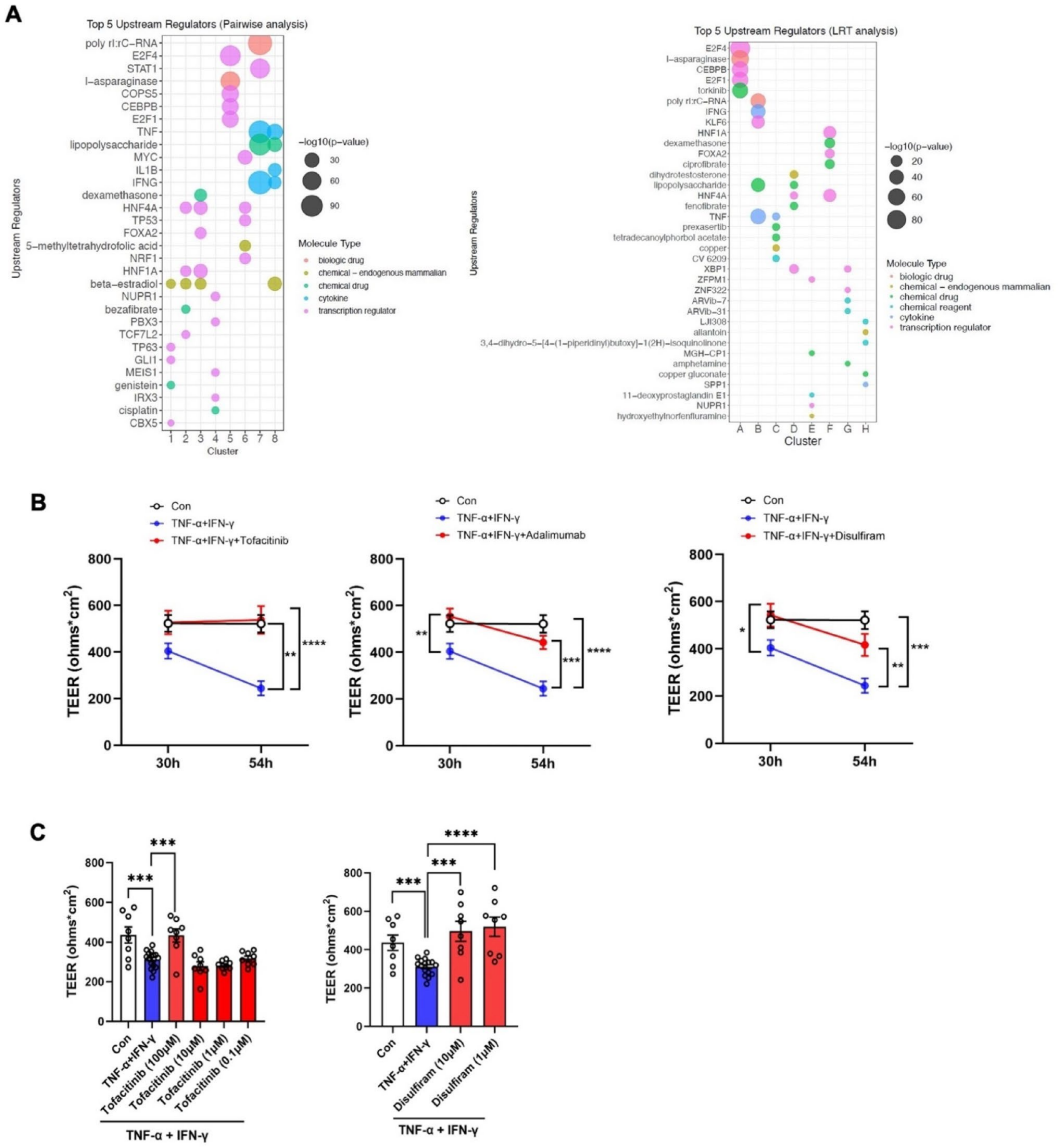
Upstream analysis of pairwise DE analysis and LRT analysis to identify the predicted molecules that could potentially reverse the transcriptome phenotypes. **A** Top 5 upstream regulators for pairwise DE analysis and LRT analysis were shown. **B** Experimental validation of predicted upstream regulators (biologic drug, chemical drug) during cytokine treatment using TEER. *N* = 4/group for control, *N* = 7 ~ 8 / group for all the other treatment group. Inhibition of core regulator or upstream regulator could reverse the intestinal barrier integrity in response to cytokine treatment at 30 h or/and 54 h. **C** TEER validation using 3D organofluic system. *N* = 8 /group except TNF-α + IFN-γ only treated group (*N* = 16 /group). The data were obtained from a single experiment with the indicated independent wells per condition. Data are presented as means ± SEM. *p-*values were determined by an ANOVA test with Sidak’s multiple comparison test for B and one-way Anova with Tukey’s multiple comparison test for C. *p < 0.05, **p < 0.01, ***p < 0.001, and ****p < 0.0001

Upstream regulator analysis was performed using the Ingenuity Pathway Analysis (IPA) upstream regulator module to predict key transcriptional regulators driving gene expression changes across the eight Caco-2 gene clusters identified from the longitudinal RNA-seq data. The resulting dataset containing regulator names, molecule types, and associated p-values for each cluster was imported into R (dplyr, tidyr, ggplot2, readr). Molecules were filtered to retain only biologically relevant categories, including transcription regulators, cytokines, growth factors, chemical reagents, chemical drugs, endogenous mammalian chemicals, and biologic drugs. This visualization (Fig. 4A) highlights distinct classes of predicted upstream regulators contributing to the transcriptional dynamics of each cluster in Caco-2 cells, offering insights into the regulatory mechanisms underlying intestinal epithelial responses.

### Crohn’s diseases/ulcerative colitis cDNA Array

TissueScan, Crohn’s and Colitis cDNA array panel I (CCRT501) and panel II which contain ten identical sets of 47–48 large and small intestine tissues covering both Crohn’s and colitis disease and normal tissue were purchased from Origene. The number of samples per group that were analyzed are as follows: non-IBD panels are 13, CD panels are 35 and UC panels are 43. Relative expression of SLC26A3 was quantified using qPCR and GAPDH was used as internal house keeping gene.

### SLC26A3 staining and quantification in human colon specimens

To evaluate and quantify the expression of SLC26A3 from CD and UC patients, the colon specimens were collected and compared with normal colon specimens. 9 normal colons, 6 CD colons and 9 UC colons were analyzed. For the tissue staining, rabbit polyclonal antibody, SLC26A3 (Invitrogen, PA5-57508), 0.25 µg/ml were used to stain the tissue and the rabbit monoclonal antibody, rabbit IgG isotype (Cell Signaling Technology, 3900 S), 0.25 µg/ml were used as control. Then Ventana Discovery Ultra autostainer was then used for autostaining. Annotations were hand drawn roughly around the transverse sections of the mucosa and muscularis mucosa then an Al algorithm was trained to separate the glass/background and submucosa from the mucosa and muscularis mucosa. Thresholds were set to separate true signal from background. The localization of SLC26A3 expression was quantified using the following formula: % Area = (µm^2^ of marker of interest / µm^2^ of mucosa & muscularis mucosa) * 100. The stained slides were digitized using an Aperio GT 450 scanner (Leica Biosystems Inc., Buffalo Grove, IL Cat #23GT450).

### Statistical analysis

Data are presented as means ± SEM. P-values were determined by an ANOVA test with Sidak’s multiple comparison test and unpaired t test (Mann-Whitney test). P-values < 0.05 were considered significant (*p < 0.05; **p < 0.01; ***p < 0.001; ****p < 0.0001). The data was analyzed using GraphPad Prism version (10.4.1).

## Results

To mimic a model of gut barrier disruption induced by inflammation, we treated human intestinal epithelial cells, Caco-2, with the key cytokines responsible for mediating inflammation including TNF-α and IFN-γ. (Fig. 1A). Caco-2 cells were cultured on transwell inserts for 12 days followed by treatment with TNF-α and IFN-γ applied to both the apical and basolateral side. Barrier integrity was assessed using transepithelial electrical resistance (TEER) and FITC-dextran leakage assays for paracellular permeability. Cytokine cocktail treatment significantly decreased TEER at 54 h (Fig. 1B). We confirmed that IFN-stimulated gene, myxovirus resistance protein 1 (*MX1*) was highly upregulated and TNF-α-inducible gene, *IL-8* was also significantly elevated throughout all time points (**Supplementary Fig. 1A**). Additionally, our bulk RNA-seq data show relatively stable expression of enterocyte differentiation marker genes across 0–54 h time course under control conditions, including brush border enzymes such as alkaline phosphatase (*ALPI*) and sucrase-isomaltase (*SI*), as well as villin (*VIL1*) and dipeptidyl peptidase-4 (*DPP4*) (**Supplementary Fig. 1B**). This stability is consistent with a maintained differentiated state over the experimental window. Upon cytokine treatment, these genes are significantly downregulated, in line with previous report [30], suggesting that inflammatory stimulation is associated with disruption of epithelial barrier differentiation and absorptive functions. Together, these observations support the presence of a differentiated enterocyte-like transcriptional state under basal conditions. A limitation is that the full differentiation trajectory (e.g. from day 1 to day 12) was not assessed, precluding direct comparison to earlier, undifferentiated states. Furthermore, we confirmed that the cell viability after the cytokine treatment at the end point was not compromised (Fig. 1C) and most of the barrier-related genes and the cell adhesion-related genes were significantly reduced at 54 h (Fig. 1D). Gene expression results for the remaining time points (6 h, 9 h, 18 h, and 30 h) are shown in **Supplementary Fig. 1C**.

Consistent with the TEER measurements, FITC-dextran permeability assays (**Supplementary Fig. 3**) further supported increased paracellular permeability under cytokine treatment. Collectively, these results support that this cytokine-mediated barrier disruption model elicits ~ 25% reduction in TEER resistance, increased paracellular permeability by more than two-fold and downregulation in some of the barrier related genes without cytotoxic effects within 54 h.

To characterize the temporal transcriptome profiles of cytokine-induced barrier disruption and inflammation in gut epithelial cells, differentiated Caco-2 cells were treated with TNF-α and IFN-γ for 6 h, 9 h, 18 h, 30 h and 54 h alongside untreated controls (0 h) and analyzed by bulk RNA sequencing. Principal component analysis (PCA) showed that the dominant separation was driven by PC1 (80% variance), consistent with a strong overall effect of cytokine exposure, whereas PC2 (11% variance) captured a smaller secondary temporal component that broadly distinguished early (6–9 h) from later (18–54 h) treated samples (Fig. 1E). Importantly, as PCA prioritizes variance rather than experimental design, the alignment of time-point separation with PC2 still supports a consistent and biologically meaningful temporal signal, despite its lower contribution to total variance. Volcano plots of DEGs at each time points were shown in **Supplementary Fig. 1D**. Pairwise differential expression (DE) analysis between cytokine treatment versus control was performed for each time point. 5,352 significantly differently expressed genes were identified and clustered based on their expression patterns. Eight total clusters were identified (cluster 1–cluster 8) and further discerned as the following groups: downregulated across most timepoints (cluster 1–3), downregulated only at early timepoints (cluster 4), upregulated across most timepoints (cluster 5–7) and upregulated only at early timepoints (cluster 8) (Fig. 2A). Additionally, canonical pathway analysis identified the enriched pathways of each cluster and top five enriched pathways for each cluster were shown in Fig. 2B. In some cases, multiple pathways share the same level of significance, resulting in more than five pathways being displayed. The ratio indicates the proportion of overlapping genes relative to the total genes associated with the pathway.

To identify the core enriched pathways from these downregulated clusters, overlapping pathways among clusters 1–3 that comprise downregulated gene sets, were analyzed and visualized (Fig. 2C). Four core pathways were significantly enriched including phase I-functionalization of compounds which involved drug/xenobiotic metabolism [31], coordinated lysosomal expression and regulation (CLEAR) signaling pathway, sphingolipid metabolism and ion channel transport. In parallel, overlapping pathways among clusters 5–7 that were characterized by upregulated gene sets, were similarly assessed. 40 core pathways were identified, and the core enriched pathways were shown in Fig. 2D.

Among the overlapping pathways from clusters 5–7, selected genes from pathways including the Rho GTPase cycle and sirtuin signaling pathway networks were further validated by qPCR analysis to capture pathway-level expression patterns identified in the RNA-seq analyses. (Fig. 2E-F). Rho-GTPase cycle network is known to regulate reactive oxygen species (*ROS*) production through nicotinamide adenine dinucleotide phosphate oxidase (*NADPH*), cytoskeleton remodeling and intestinal inflammation [23], while sirtuin signaling network regulates cell metabolism [32], regulates oxidative stress [33] and protects against chronic inflammation [34]. The results demonstrated that these genes were significantly upregulated by cytokine treatments at indicated time points which is consistent with the RNA-seq temporal regulation pattern. This suggests that our bulk RNA-seq analysis is robust to capture the gene expression temporal changes validated by qPCR.

In parallel with pairwise DEG comparisons, we used a likelihood ratio test (LRT) time course analysis to identify genes with significant expression changes across the entire time series, allowing us to detect dynamic patterns that pairwise tests alone could have missed. We identified eighteen clusters (**Supplementary Fig. 2**) characterized by distinct cytokine-mediated temporal patterns. The eighteen clusters were subsequently grouped based on similarities of their temporal patterns and then further consolidated into ten major-clusters (cluster A–cluster J) (Fig. 3A). Due to the limited number of DE genes identified in the cluster I and J, these two clusters were not included for the following pathway enrichment analyses. The eight major- clusters were grouped as upregulated across all time points (cluster A, B, C), early and transiently upregulated (cluster D), downregulated across all time points (cluster E, F, G), and early and transiently downregulated (cluster H). The top five enriched pathways of these eight clusters were shown in Fig. 3B. To identify the core enriched pathways from LRT analysis, overlapping clusters among A-C which consisted of upregulated gene sets and overlapping clusters within cluster E-G that are considered downregulated gene sets were analyzed. Interestingly, we identified two overlapping core pathways among cluster A-C as the RHO GTPase cycle pathway which is consistently identified by pair-wise DE analysis along with the neutrophil degranulation pathway. In contrast, the synaptogenesis signaling pathway was uniquely shared among cluster E-G (Fig. 3C).

Given that the tight junction signaling pathway could be found between cluster B and C among five pathways whereas the non-canonical WNT pathway was identified among cluster A and B among fifty-two pathways (Fig. 3D) and their relevance in gut barrier integrity, we performed qPCR analysis for selected genes identified from these two pathway networks (Fig. 3E, F). Since tight junction signaling pathway identified in both cluster B and cluster C reflect key aspects of epithelial barrier functions along with cell junction organization pathway and the regulation of the epithelial-mesenchymal transition (EMT) in the development pathway, we integrated these three pathways and referred as the epithelial barrier remodeling network. The qPCR analysis of selected genes that comprising claudin 1 (*CDLN1*), f11 receptor (*F11R*) also called junctional adhesion molecule-A (JAM-A), laminin subunit gamma 2 (*LAMC2*), myosin X (*MYO10*), β-catenin (*CTNNB1*) and tumor necrosis factor receptor superfamily member 11b (*TNFSF11B*) confirmed the temporal cytokine response pattern identified in LRT analysis (Fig. 3E).

In addition, the non-canonical Wnt pathway activates the Wnt/planar cell polarity (PCP) pathway which regulates cellular orientation and polarity as well as cytoskeletal remodeling and Wnt/Ca^2+^ signaling cascade, which play a critical role in regulating self-renewal, proliferation and differentiation [35]. Genes identified in this network including proteasome activator complex subunit 1 (*PSME1*), phophoinositide 3-kinase (*PI3K*) regulatory subunit 3 (*PIK3R3*), wnt family member 4 (*WNT4*), Rac family small GTPase 1 (*Rac1*), SMAD specific E3 ubiquitin protein ligase 1 (*SMULF1*) and serine/threonine kinase 31 (*STK31*) were analyzed by qPCR and confirmed the expression pattern from LRT analysis (Fig. 3F). The selected genes from both epithelial barrier remodeling and non-canonical WNT pathway were significantly elevated in cytokine treated group except *STK31*.

To identify the potential upstream regulators that could be modulating the temporal gene expression responses to cytokine-induced gut barrier disruptions, we performed an upstream regulator analysis on the genes from pairwise DE analysis and LRT analysis. The top five upstream regulator molecules based on significance were visualized in each cluster in both pairwise DE analysis and LRT analysis (Fig. 4A) and all upstream regulator molecules enriched in each cluster are shown in **Supplementary Data 3 and 4.**

Among all upstream regulators, several predicted upstream regulators including janus kinase 2 (*JAK 2*), *TNF* and the chemical, disulfiram [36] were further selected for functional validation assays. We examined the effect of JAK2 inhibitor, *TNF* inhibitor and disulfiram to assess their effects on cytokine-induced barrier disruption in differentiated Caco-2 cells. Interestingly, co-treatment of tofacitinib (a JAK1/2 inhibitor), adalimumab (a *TNF*-neutralizing antibody) and disulfiram (a gasdermin D inhibitor) exerted a resistance against cytokine-induced epithelial barrier disruption measured by consistent TEER values (Fig. 4B) and reduction of the intestinal permeability (**Supplementary Fig. 3A**). We observed that beta estradiol, another predicted upstream regulator, also enhanced intrinsic barrier integrity by significantly increasing TEER in cytokine-treated conditions and reducing intestinal permeability (**Supplementary Fig. 3B**). To further investigate the role of some of the upstream regulators under more physiologically mimicking conditions, we assessed the effects of tofacitinib and disulfiram using a 3D microfluidic OrganoPlate system. Treatment with tofacitinib at 100 µM preserved epithelial barrier integrity upon cytokine treatment as indicated by significant increase in TEER values at 54 h restoring levels comparable to the untreated control. Similarly, disulfiram treatment at 10 µM and 1 µM also conferred protective effects in cytokine treated Caco-2 cells on the epithelial barrier at 54 h by restoring TEER to the levels of untreated control (Fig. 4C).

One of the predicted upstream regulators is hepatocyte nuclear factor 4 alpha (HNF4α), which is a transcription factor that governs the expression of numerous genes involved in hepatic function, lipid metabolism and intestinal development. Emerging evidence has implicated that variants in the *HNF4A* locus are associated with increased IBD susceptibility [37]. Interestingly, we identified HNF4α as an upstream regulator through both pairwise DE and LRT analysis. However, treatment of HNF4α agonist, n-trans-caffeoyltyramine (NCT) [38] with cytokine treatment did not rescue the epithelial barrier integrity and significantly increased permeability (**Supplementary Fig. 3C**) in our differentiated Caco-2 barrier-disruption model.

**Fig. 5:**
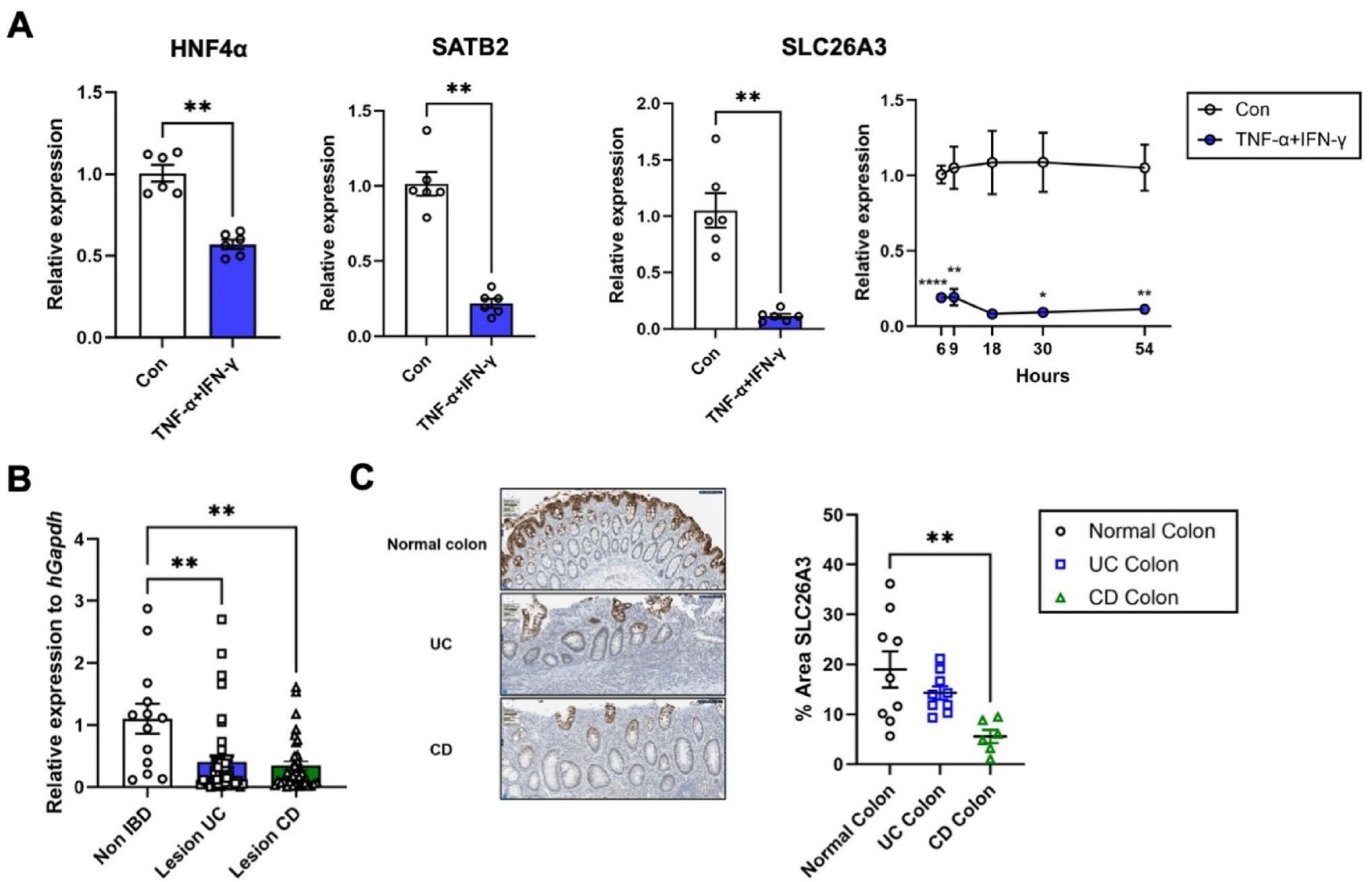
*HNF-4α*regulating gene, *SLC26A3*was significantly down-regulated in IBD patient colon tissue as well as in Caco-2 cells.**A** Relative expression of *HNF4α*, *SATB2* and *SLC26A3* at 54 h in cytokine treated Caco-2 cells and *SLC26A3* expression in each time point. *N* = 6 /group. **B** Relative expression of *SLC26A3* in non-IBD colon tissue (*N* = 13), colon lesion from UC patient and (*N* = 43) and colon lesion from CD patient (*N* = 35) using Crohn’s/colitis cDNA array. **C** The area of SLC26A3 in normal colon tissue (*N* = 9), UC (*N* = 9) and CD (*N* = 6) colon tissues were quantified. Caco-2 qPCR data were derived from two independent experiments with N representing replicate wells per condition. For human specimens, N represents biological replicates. Data are presented as means ± SEM. *p-*values were determined by an unpaired *t*-test for (A) Kruskal–Wallis test with Dunn’s multiple comparison test for (**B**) For C, the scale bar indicates 100 µM at 20x magnification and ANOVA test with Tukey’s multiple comparison test was used. *p < 0.05, **p < 0.01, ***p < 0.001, and ****p < 0.0001

Among its downstream targets, HNF4α transcriptionally regulates solute carrier family 26 member 3 (SLC26A3), a chloride/bicarbonate exchanger critical for maintaining fluid and electrolyte homeostasis in the intestinal epithelium. Beyond its canonical transporter role, a recent study suggests a potential role for SLC26A3 in supporting intestinal barrier integrity [39]. Notably, mice deficient in downregulated in adenoma (DRA), the protein encoded by *SLC26A3*, exhibit impaired epithelial barrier function and spontaneous colitis underscoring its potential contribution to IBD pathophysiology. Here, we found that *SLC26A3*, *HNF4A* and special AT-rich sequence-binding protein 2 (*SATB2*), a chromatin organizer that spatially restricts HNF4α binding were all significantly downregulated in differentiated Caco-2 cells after the cytokine exposure at 54 h (Fig. 5A). We found that *SLC26A3* was rapidly downregulated as early as 6 h following cytokine treatment and remained suppressed through 54 h (Fig. 5A) which also supports the previous studies [40–42]. Next, we obtained the cDNA array panels consisting of the small and large intestine of Crohn’s and Ulcerative Colitis patients and quantified the *SLC26A3* expression levels compared to adjacent lesion of normal tissues. Consistent with our observation in differentiated Caco-2 barrier-disruption model, *SLC26A3* was significantly downregulated in lesions of CD and UC patients (Fig. 5B). We further examined the SLC26A3 protein expression in normal and diseased human colon using histological analysis. In agreement with our mRNA expression results, the colon histological data for CD and UC patients validated significant downregulation of *SLC26A3*. The abundance of SLC26A3 showed a significant decrease in CD and UC colon epithelium compared to the normal colons (Fig. 5C) although these reductions are statistically different in CD compared to the normal colons.

## Discussion

TNF-α and IFN-γ are well-established as central proinflammatory cytokines driving epithelial barrier dysfunction in IBD [43]. Prior studies have demonstrated a synergistic effect of these cytokines in disrupting epithelial integrity [44,45]. Notably, TNF-α induced barrier dysfunction was only evident on prior exposure to IFN-γ, whereas pre-incubation with TNF-α followed by IFN-γ did not elicit increased permeability [44]. Also, despite IFN-γ enhancement of TNFR1 and TNFR2 expression on the basolateral membrane of epithelial cells, only TNFR2 signaling mediates TNF-α-induced barrier disruption [45]. Although TNF-α and IFN-γ co-treatment can induce cytotoxicity through non-canonical TNFR1-mediated apoptosis or necroptosis involving the *JAK1/2*–*STAT1*–*CASP8* axis [46], our results confirm that within a 54-hour window, co-incubation of these cytokines significantly impaired barrier function in differentiated Caco-2 cells without affecting cellular viability.

Temporal transcriptomic profiling revealed a clear bifurcation between early and late transcriptional responses, implicating distinct molecular programs in the progression of cytokine-induced epithelial injury. Both likelihood ratio test (LRT) and pairwise differential expression (DE) analyses identified the Rho GTPase cycle as a key enriched pathway.

Our findings indicate significant enrichment of Rho-regulated genes associated with cytoskeletal remodeling and oxidative stress. For example, *NCF2* and *NOX1*, key mediators of *Rac2*- and *Rac1*-dependent ROS production, were markedly upregulated, indicating enhanced oxidative signaling that may contribute to epithelial stress and barrier dysfunction [47,48]. *IQGAP3*, a scaffold protein that promotes actin polymerization [49] and *BAIAP2L2*, which regulates membrane curvature and actin dynamics [50], were also increased, pointing to active cytoskeletal remodeling and heightened cellular plasticity. This is further supported by the upregulation of *RND1*, an atypical Rho GTPase that weakens cell-cell adhesion, was elevated, consistent with reduced epithelial integrity [51]. Notably, *JAG1*, a Notch ligand involved in epithelial differentiation and homeostasis, was also induced, suggesting activation of Notch signaling as a potential compensatory response to epithelial injury. Together, these findings support a model in which Rac-driven ROS production and Rho-mediated cytoskeletal reorganization converge to promote barrier disruption, with concurrent engagement of Notch signaling pathways potentially acting to restore epithelial homeostasis.

Rho GTPases are known to regulate multiple downstream pathways that control cytoskeletal dynamics and epithelial barrier function, and their dysregulation has been implicated in intestinal inflammation and epithelial dysfunction. In the context of IBD, however, their role appears to be highly context dependent. For example, RhoA signaling may contribute to maintaining barrier homeostasis, whereas excessive activation, particularly via the RhoA/ROCK pathway, has been associated with increased inflammation and barrier dysfunction [23]. Thus, the observed upregulation of Rho signaling may reflect either a compensatory protective response or a pathogenic mechanism. While we did not directly assess the effects of inhibiting Rho-associated protein kinase (ROCK), a key downstream effector of RhoA, in our barrier-disruption model, a previous report has shown that ROCK inhibitor, Y-27,643, can attenuate ethanol induced barrier disruption in Caco-2 [52], suggesting the potential effects of Rho GTPases pathway in regulating barrier integrity.

Sirtuins (NAD⁺-dependent deacetylases) are key regulators of metabolism, oxidative stress, barrier function, and immunity [53,54]. In this study, cytokine exposure led to enrichment of the sirtuin signaling pathway, accompanied by upregulation of several sirtuin-associated genes (*SLC2A1*, *NAMPT*, *NOS2*, *MYC*, *SOD2*, and *DUSP6*). However, several changes suggest disrupted sirtuin control under inflammation. For instance, GLUT1 (*SLC2A1*), normally suppressed by SIRT1/6, was increased, indicating loss of inhibition [55,56]. *NAMPT*, a key NAD⁺ biosynthetic enzyme, was also strongly induced, consistent with colitis models, although its inhibition can be protective in a SIRT6-dependent manner [57,58]. While *NOS2*-derived nitric oxide promotes inflammatory and nitrosative stress, sirtuins, particularly SIRT1, counteract these effects by suppressing NF-κB activity and supporting cellular homeostasis [59]. In addition, the marked induction of *MYC*, a transcription factor regulated by SIRT1 and SIRT6, further supports its role in promoting proliferation and sustaining inflammatory signaling, potentially contributing to barrier dysfunction [60]. In contrast, the sustained upregulation of *SOD2*, a mitochondrial antioxidant enzyme activated by SIRT3, may represent a compensatory response aimed at mitigating oxidative stress.

A recent study demonstrates that pharmacologic SIRT2 inhibition strengthens intestinal epithelial barrier function and protects mice from experimental colitis [54]. Together with the reports [61,62] that *DUSP6*-deficient Caco-2 monolayers exhibit enhanced TEER and induced selected TJP genes, these data suggest a literature-based hypothesis that SIRT2 blockade may reduce the amplitude/duration of the early *ERK*-*DUSP6* feedback burst, thereby limiting the permissive window for junctional destabilization. These findings suggest that during cytokine-driven barrier injury, increased expression of genes associated with the sirtuin axis may be linked to two complementary processes: enhanced epithelial protection and selective inflammatory responses that aid in resolution.

Collectively, these observations position sirtuins as higher-order regulators of metabolic and inflammatory networks whose disruption may exacerbate epithelial damage and accelerate disease.

In addition to pair-wise DE analysis, our temporal transcriptome analysis highlighted two other key pathways: epithelial barrier remodeling and non-canonical WNT signaling. The epithelial barrier remodeling network which comprises of tight junction organization, cell junction regulation, and epithelial–mesenchymal transition (EMT) was validated. Notably, *CLDN1*, F11R (*JAM-A*), *LAMC2*, and *TNFRSF11B* were significantly upregulated across all timepoints following cytokine stimulation, implicating in their potential compensatory mechanisms in junctional remodeling and tissue repair processes under inflammatory stress.

In contrast, *MYO10* and *CTNNB1* (β-catenin) displayed a biphasic expression pattern which was initially upregulated at early timepoints but subsequently downregulated during later timepoints. This dynamic suggests that while early activation may support cytoskeletal tethering and adhesion, the later decline reflects a breakdown in adhesive integrity and cytoskeletal coordination, contributing to progressive barrier disruption.

The non-canonical WNT signaling pathway, which operates independently of β-catenin, was also enriched in our analysis and has been implicated in both wound healing and disruption of epithelial polarity. We observed upregulation of *PSME1*, *PIK3R3*, *WNT4*, *RAC1*, *SMULF1* and *STK31* suggesting a coordinated and dynamic activation of pathways that promote epithelial plasticity and remodeling. Notably, *STK31*, previously reported to control the differentiation state of colon cancer cell [63], remained largely unchanged, highlighting potential pathway selectivity in the epithelial response to cytokine stimulation. *WNT4* and *RAC1* act as central drivers of cytoskeletal reorganization and wound closure [64,65], while *PIK3R3* amplifies PI3K-dependant signaling that intersects with *Rac1* activity [66]. *SMURF1* modulates RhoA stability, providing a layer of ubiquitin-mediated control over polarity and adhesion [67]. Although *PSME1* functions more indirectly, its role in proteasome-mediated turnover of signaling molecules suggests it may influence the balance between barrier repair and chronic inflammation [68].

In this study, six representative genes were selected for RT–qPCR validation based on their relevance to the most significantly enriched pathways identified in the pairwise DEG analysis and LRT analysis. The selected genes include key representatives of the major pathways and capture the overall expression trends observed in the RNA-seq dataset. Importantly, the RT–qPCR results showed concordant expression patterns with the RNA-seq data, supporting the robustness and reproducibility of our findings. Given that our primary conclusions are based on pathway-level enrichment rather than individual gene effects, we prioritized the validation of representative genes from key pathways rather than increasing the number of genes.

Both the pair-wise differential expression and likelihood ratio analyses identified *HNF4A* as a potential upstream regulator. This finding is aligned with prior studies reporting reduced HNF4α expression in individuals with IBD [69], as well as loss of HNF4α disrupts epithelial–immune interactions, weakens barrier–immune homeostasis, and increases susceptibility to colitis [70]. In line with these observations, we found that several well-established HNF4α target genes, including *SATB2* and *SLC26A3*, were markedly downregulated in cytokine-treated differentiated Caco-2 cells and in colonic tissues from patients with IBD, further supporting the role of HNF4α in maintaining epithelial homeostasis and barrier integrity. However, they do not establish that *SLC26A3* downregulation is itself the direct cause of barrier loss in our system and dedicated gain- and loss-of-function studies will be needed to test that hypothesis. Moreover *SLC26A3* has been reported to be involved in stimulating baseline mucus expansion in the distal colon by regulating apical bicarbonate secretion and the luminal ionic environment, which is required for post-secretory mucin unfolding and expansion [71]. While we observed downregulation of *SLC26A3* in both in vitro and patient-derived samples under inflammatory conditions, our findings are associative and do not establish a direct causal role of *SLC26A3* in regulating epithelial barrier integrity and further studies are required.

NCT, as a compound that can enhance HNF4α activity, has been shown to enhance the gut barrier function and reduce gut permeability when treated in human primary intestinal cells treated with TNF-α [72]. However, in our differentiated Caco-2 model, NCT did not confer such protection to TNF-α/IFN-γ-induced barrier disruption (**Supplementary Fig. 3C**). The discrepancy may reflect differences in inflammatory severity, as TNF-α/IFN-γ synergistically induces a stronger barrier defect than low-dose TNF-α alone.

Although *HNF4A* was significantly downregulated under inflammatory conditions (Fig. 5A), whether NCT effectively activates HNF4α downstream target genes in Caco-2 cells remains unknown. In addition, since it is still unclear whether NCT improves barrier function in human gut epithelial cells through direct induction of HNF4α expression, reduced HNF4α abundance may limit the extent to which NCT can agonize HNF4α and restore barrier integrity. Moreover, even if HNF4α is present and activated, its function may depend on appropriate chromatin context. For instance, HNF4α may require cooperation with chromatin organizers such as SATB2 to fully regulate its target genes, and loss of SATB2 during inflammation could further limit HNF4α access to these loci.

In addition, differences between primary intestinal epithelial cells and transformed Caco-2 cells may influence HNF4α target engagement and downstream barrier responses. Together, these factors may render HNF4α agonism insufficient to restore barrier function in this context.

Using this differentiated Caco-2 gut barrier-disruption model, our temporal transcriptome analysis nominated several actionable upstream regulators including *JAK2*, gasdermin D, and *TNF*, along with β-estradiol (Fig. 4, **Supplementary Fig. 3A**,** B**) and perturbing these nodes mitigated barrier disruption in differentiated Caco-2 monolayers, directly linking transcriptomic signals to functional protection. Importantly, JAK inhibitors and TNF-α antagonists, both approved therapies for IBD, attenuated barrier disruption in our epithelial-only system, indicating that key aspects of cytokine-driven barrier dysfunction are recapitulated in this model. In practical terms, the model reveals druggable epithelial control points and can forecast therapeutic response timing, while remaining standardized, scalable, and genetically tractable that is well suited for timing experiments and medium-throughput prioritization before escalation to immune-competent co-cultures, organoids, or gut-on-chip platforms.

In our cytokine-induced barrier disruption model, barrier integrity loss occurred without affecting cell viability. Disulfiram partially rescued the barrier defect, suggesting that gasdermin D/inflammasome-associated inflammatory membrane injury may contribute to barrier dysfunction, potentially through non-lethal sublytic membrane pore formation rather than overt pyroptotic cell death [73,74].

While Caco-2 Transwell models are widely used to study the intestinal barrier, their tumor-derived background limits their representation of normal intestinal epithelium, and they do not capture the multicellular and biomechanical complexity observed in more advanced systems (e.g., gut-on-a-chip) [75]. We therefore interpret our findings within epithelium-intrinsic programs, and this focus may underrepresent genes influenced by immune, stromal, or microbial cues, but it nonetheless enabled high-resolution mapping of junctional integrity, cytoskeletal dynamics, and transport processes that are directly relevant to barrier failure and repair.

We also note that, our IBD gut barrier model relies on a single fixed-dose cytokine cocktail, which does not reflect the dynamic, context-dependent nature of the in vivo environment [76], thereby limiting physiological relevance. The TNF-α concentration used here (100 ng/mL) exceeds physiological levels but is consistent with commonly used in vitro conditions designed to induce robust epithelial inflammatory responses. Another limitation is that the cytokine cocktail was not replenished during the 54-hour treatment, and its effective concentration may decrease over time due to thermal and biochemical instability. Accordingly, later time points likely reflect cellular responses to a time-evolving exposure rather than a constant cytokine dose. Despite this, a robust barrier phenotype was observed at 54 h without evidence of cytotoxicity, indicating sustained biological activity under these conditions. TNF-α and IFN-γ induce rapid transcriptional responses, with many immediate-early targets activated within 1–2 h. As the present time course begins at 6 h following combined cytokine treatment, it does not capture this initial phase. Instead, the transcriptional changes captured here reflect integrated downstream and sustained programs arising from concurrent TNF-α and IFN-γ signaling, including secondary regulatory responses. Notably, while early transcriptional activation is rapid, functional outcomes such as epithelial barrier disruption typically require prolonged exposure (24–72 h). Accordingly, the temporal design of this study is aligned with capturing transcriptional dynamics associated with these later functional phenotypes rather than the immediate-early response. In addition, all experiments were conducted in a Caco-2 clone stably expressing Cas9, and interpretations are therefore restricted to this background. An advantage of this system is that it enables direct CRISPR-based perturbation of candidate regulators identified from the time-course data, providing a tractable route for future functional validation, which is beyond the scope of the present study.

In this context, a further constraint of this study is the relatively small number of independent biological replicates for the RNA-seq time course, with variable representation across time points. As a result, the analysis emphasizes reproducible temporal patterns and pathway-level changes rather than relying solely on statistical significance at the individual gene level. Future studies with increased replication will be important to further validate these findings.

Methodologically, combining pairwise differential expression with an LRT-based time-course analysis was essential. Both approaches converged on the Rho GTPase cycle as a key pathway, controlling cytoskeletal regulation and epithelial dynamics, while each approach uncovered complementary pathway sets with distinct temporal sensitivities, the features that would be obscured by static contrasts alone. We reinforced these bioinformatic predictions by qPCR validation of representative genes and by upstream regulator analysis identifying HNF4α as a key epithelial transcriptional hub and downstream targets such as *SATB2* and *SLC26A3* were cohesively modulated in differentiated Caco-2 cells and in colonic tissues from patients with IBD. Together, our study establishes a coherent, time-resolved regulatory architecture in a differentiated Caco-2 barrier-disruption model, nominates tractable intervention points (e.g., *JAK2*, *GSDMD*, *TNF* modulation), and generates mechanistic hypotheses testable in higher-order systems. By delineating when, not just whether specific pathways are perturbed, our study advances a generalizable framework for timing-aware therapeutic interrogation of epithelial barrier failure and strengthens the translational relevance of our findings. To our knowledge, this is among the first epithelium-only, cytokine-challenge, time-course RNA-seq maps in differentiated Caco-2 cells, beyond TEER/qPCR endpoints and static single-timepoint omics. This temporal framework suggests candidate early (6–9 h) and late (18–54 h) phases of gut epithelial injury. The early phase is characterized by rapid signaling and remodeling responses, whereas the later phase reflects sustained barrier compromise together with suppression of homeostatic epithelial genes. Across pathways, these dynamics are heterogeneous: some programs (e.g., epithelial barrier remodeling) are predominantly early, while others (e.g., sirtuin signaling and cell cycle-related pathways) include both early-responsive and sustained components. Framing the data in this temporal context provides a conceptual basis for identifying phase-specific pathway engagement and potential intervention windows, based on the time-resolved transcriptomic and barrier trajectory, rather than implying definitive therapeutic assignment.

## Supplementary Information

Below is the link to the electronic supplementary material.

Supplementary Material 1

Supplementary Material 2

Supplementary Material 3

Supplementary Material 4

Supplementary Material 5

## Data Availability

RNA-seq data from this study was deposited in the GEO database with accession number GSE314628.
