# Supplementary material for: Temporal transcriptomic profiling identifies core regulators in cytokine-induced gut barrier disruption in differentiated Caco-2 cells
Source: Inflamm Res. 2026 Jun 8;75(1):135. doi: 10.1007/s00011-026-02288-5 (PMC13246533; doi:10.1007/s00011-026-02288-5)
Supplement: Supplementary file 5 — Supplementary Material 5 [file 11_2026_2288_MOESM5_ESM.docx]

**Supplementary figures**

**Supplementary figure 1**

**
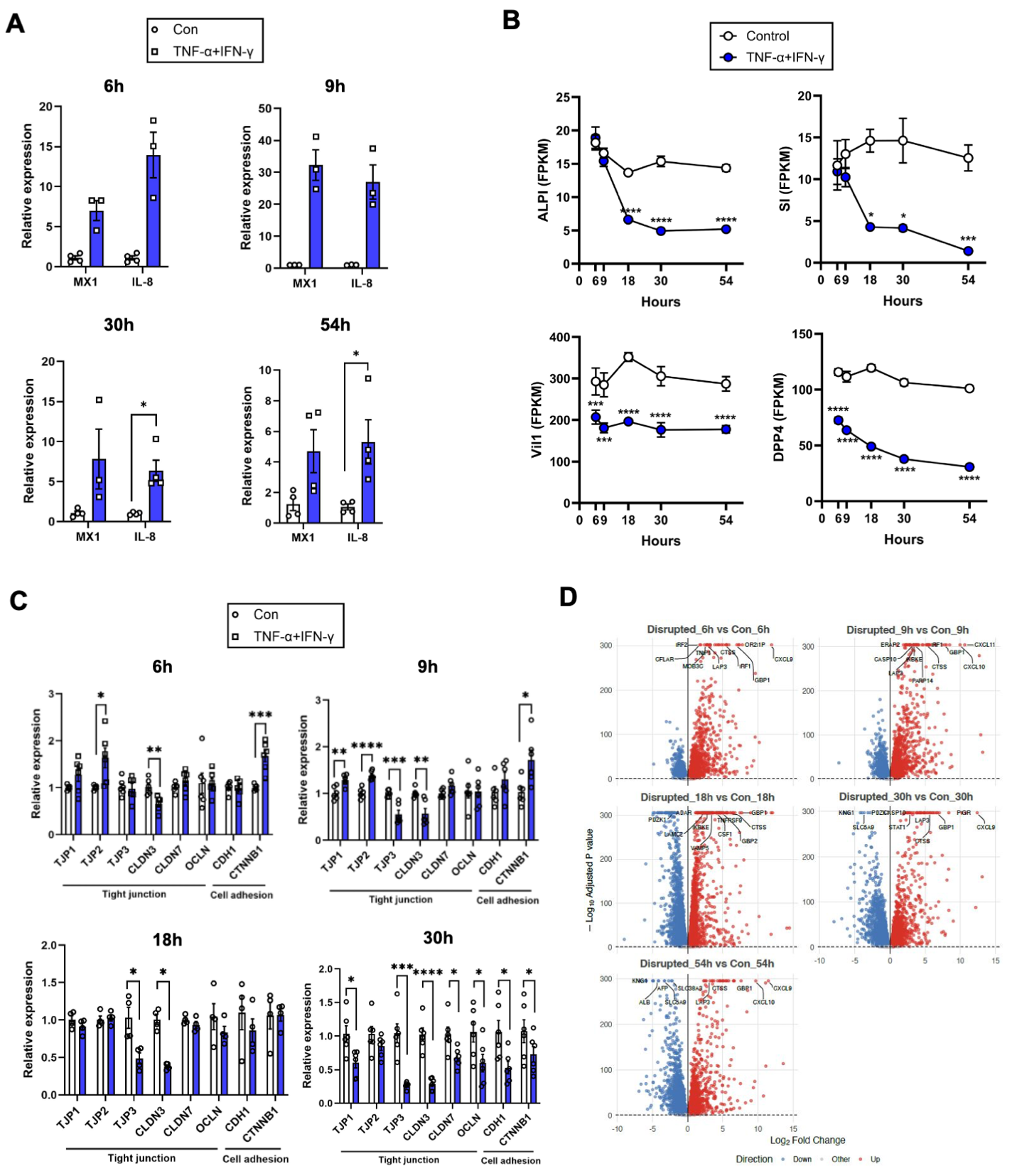
**

**Supplementary Figure 1.** (A) Relative expression of MX1 and IL-8 after the cytokine treatment (N=3~4/group). (B) Normalized expression of Caco-2 differentiation markers in FKPM using bulk RNA-seq datasets. (C) Relative expression of tight junction related genes and cell adhesion genes at 6h, 9h, 30h and 54h (N=5~6/group except the control group at 18h (N=4). (D) Volcano plots of log_2_ fold changes of differentially expressed genes at each time point (6h, 9h, 18h, 30h and 54h). Bulk RNA-seq and qPCR data were derived from two independent experiments and (D) was collected from single experiment. The number of replicates is indicated for each experiment. Data are presented as means ± SEM. *p*-values were determined by an ANOVA test with Sidak’s multiple comparison test for B. *p*-values were determined by unpaired t-test for C and Mann-Whitney U test for D. **p* < 0.05, ***p* < 0.01, ****p* < 0.001, and *****p* < 0.0001.

**
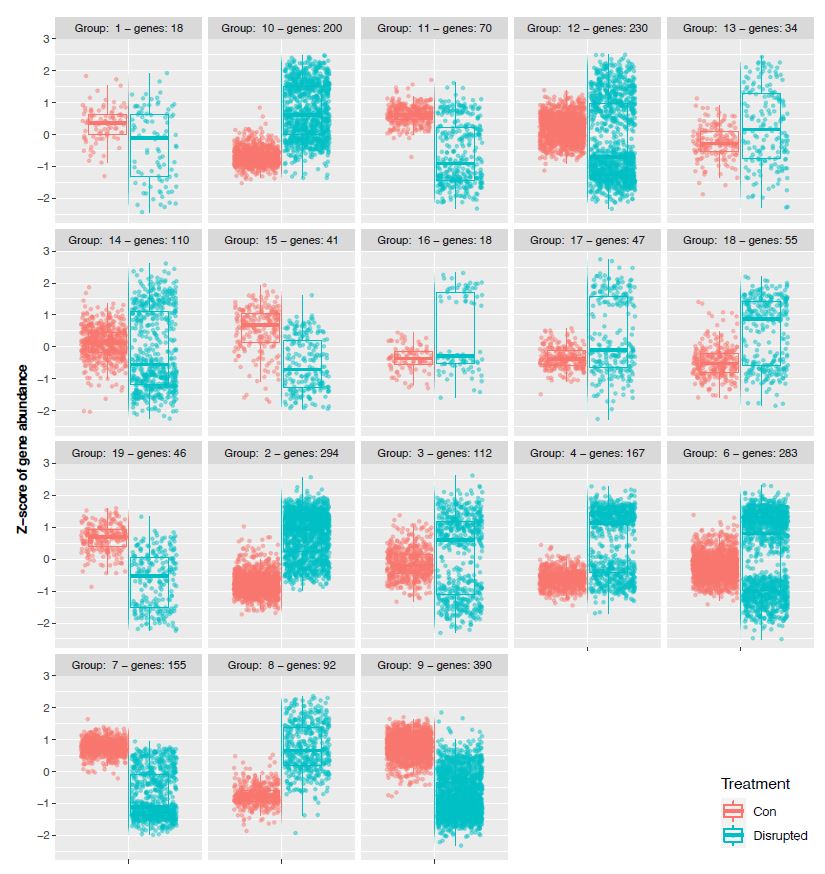
Supplementary Figure 2**

**Supplementary Figure 2.** LRT analysis identified the 18 gene clusters showing particular patterns across samples between control and cytokine treated group. Z-score of gene abundance of each cluster were shown.

**
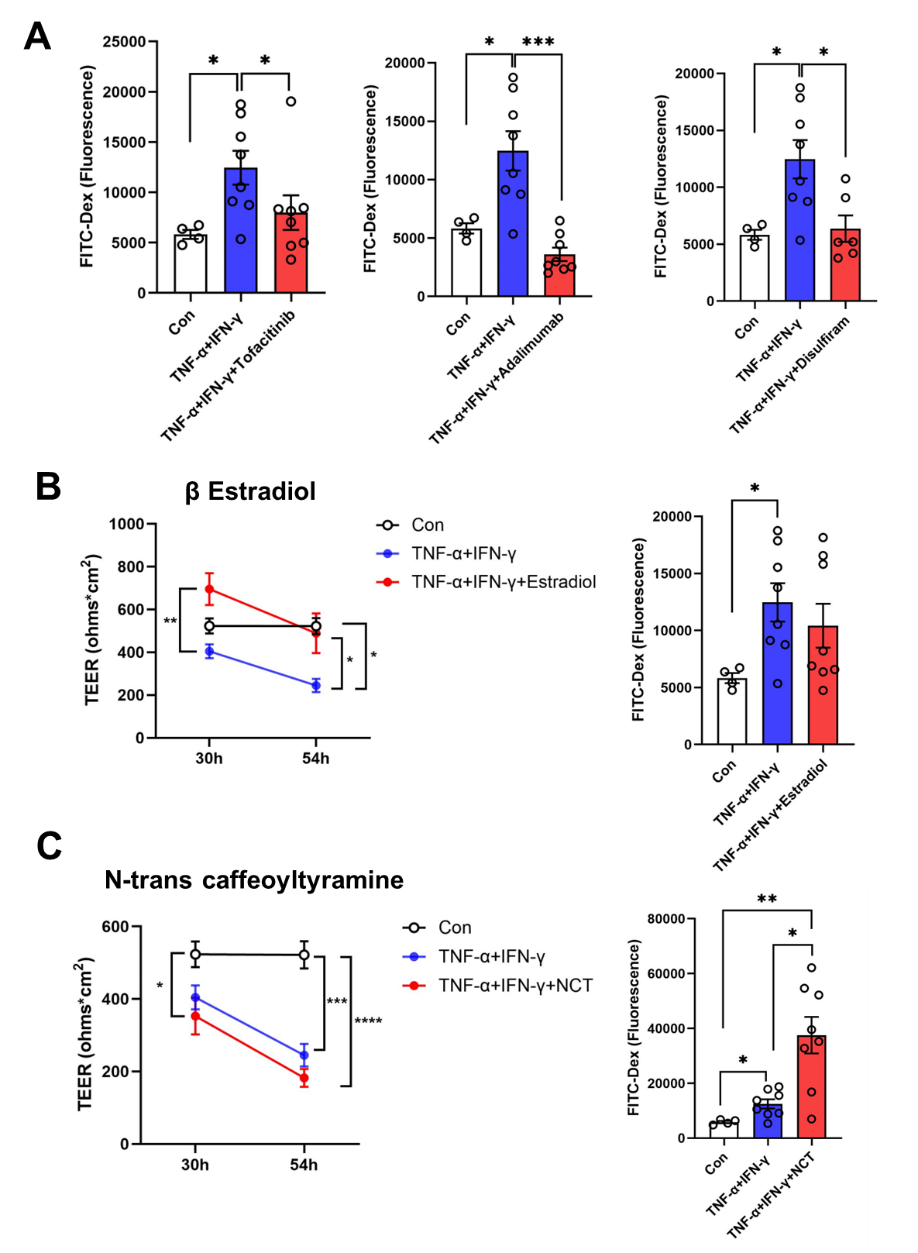
Supplementary Figure 3**

**Supplementary Figure 3. Some selected upstream regulators identified in cytokine treated Caco-2 cells were able to restore the barrier permeability when co-treated with cytokines.**  (A) Tofacitinib (100 μM), adalimumab (1 μg/ml), or disulfiram (10 μM) were co-treated with mixtures of cytokines (TNF-α, IFN-γ) and FITC-Dex permeability assay was performed at 54h. (N=6~8 /group except control: N=4 /group). The control group is treated with 0.1% DMSO. (B) β Estradiol (10 μM), (C) N-trans-caffeoyltyramine (NCT) (50 μM) were co-treated with mixtures of cytokines (TNF-α, IFN-γ) and TEER was measured at 30h and 54h. (N=8 /group, control group; N=4 /group). FITC-Dex permeability assay were performed at 54h. (N=6~8 /group, control group; N=4 /group). The control group is treated with 0.1% DMSO. Results were obtained from a single experiment with the indicated replicate wells per condition. Data are presented as means ± SEM. Data are presented as means ± SEM. *p*-values were determined by Mann-Whitney U test for A, (B, right) and (C, right). *p-*values were determined by an ANOVA test with Sidak’s multiple comparison test (B, left) and (C, left). **p* < 0.05, ***p* < 0.01, ****p* < 0.001, and *****p* < 0.0001.

**Table S1. TaqMan probes used in the study.**

| **Gene** | **TaqMan Assay ID** |
| --- | --- |
| *TJP1* | Hs01551871_m1 |
| *TJP2* | Hs00910543_m1 |
| *TJP3* | Hs00274276_m1 |
| *CLDN3* | Hs00265816_s1 |
| *CLDN7* | Hs00600772_m1 |
| *OCLN* | Hs05465837_g1 |
| *CDH1* | Hs01023895_m1 |
| *CTNNB1* | Hs00355045_m1 |
| *SLC26A3* | Hs00995363_m1 |
| *SATB2* | Hs01546836_m1 |
| *PDZD3* | Hs01084723_g1 |
| *HNF4α* | Hs01071345_m1 |
| *NCF2* | Hs01084940_m1 |
| *NOX1* | Hs01071088_m1 |
| *IQGAP3* | Hs00603642_m1 |
| *BAIAP2L2* | Hs01052417_m1 |
| *RND1* | Hs00262808_m1 |
| *JAG1* | Hs01070032_m1 |
| *SLC2A1* | Hs00892681_m1 |
| *NAMPT* | Hs00237184_m1 |
| *NOS2* | Hs01075529_m1 |
| *MYC* | Hs00153408_m1 |
| *SOD2* | Hs00167309_m1 |
| *DUSP6* | Hs04329643_s1 |
| *CLDN1* | Hs00221623_m1 |
| *F11R* | Hs00375889_m1 |
| *LAMC2* | Hs01043717_m1 |
| *MYO10* | Hs05592463_s1 |
| *CTNNB1* | Hs00355045_m1 |
| *TNFRSF11B* | Hs00900358_m1 |
| *PSME1* | Hs00389209_m1 |
| *PIK3R3* | Hs01103591_m1 |
| *WNT4* | Hs01573505_m1 |
| *RAC1* | Hs01902432_s1 |
| *SMURF1* | Hs00410929_m1 |
| *STK31* | Hs00230012_m1 |

**Table S2. The list of the chemicals/drugs used in the study.**

| **Chemicals/Drugs** | **Vendor** | **Cat number** | **Treatment concentration** |
| --- | --- | --- | --- |
| β-estradiol | Medchemexpress | HY-B01411G | 10 μM |
| Resveratrol | Medchemexpress | HY-16561 | 10 μM |
| Disulfiram | Medchemexpress | HY-B0240 | 10 μM |
| Tofacitinib | Medchemexpress | HY-40354 | 100 μM |
| Adalimumab solution | Sigma-Aldrich | A-166 | 1μg/ml |
| N-trans-caffeoyltyramine (NCT) | Medchemexpress | HY-N8241 | 50 μM |
